# Super active surveillance for low-risk prostate cancer | *Opinion: Yes*

**DOI:** 10.1590/S1677-5538.IBJU.2019.02.02

**Published:** 2019-04-01

**Authors:** Leonardo O. Reis, Danilo L. Andrade, Fernando J. Bianco

**Affiliations:** 1 UroScience, Pontifícia Universidade de Campinas - PUC Campinas, Campinas, SP, Brasil;; 2 Departamento de Urologia, Universidade Estadual de Campinas - UNICAMP, Campinas, SP, Brasil;; 3 Urological Research Network, Miami, FL, USA

**Keywords:** Prostatic Neoplasms, Risk Reduction Behavior, Watchful Waiting, Therapeutics

Prostate cancer is the most common solid tumor in men in western countries. Notwithstanding, its high incidence, most patients survive their prostate cancer diagnosis and die from other causes (1). This low cancer death event rate poses remarkable challenges for both patients and their treating physicians. Fundamentally the “overs”, meaning overdiagnosis and overtreatment (2). Both particularly important as significant issues for patients arise as consequences of treatment. Distastefully, urinary incontinence and erectile dysfunction, among other, both exerting substantial impact in quality of life (3).

This decade has witnessed results from three randomized trials. These robust studies clearly pointed to a limited benefit of definitive intervention such as surgery or radiation vs. surveillance modalities. The lack of differences in all cause survival and the relative low rate of metastasis 10 and 15 years after diagnosis have changed dramatically our knowledge on what is best to do when a man presents with a newly diagnosed prostate cancer (4–6).

Not surprisingly, active surveillance (AS) has become a definitive alternative and common option. This strategy of management certainly decreased the morbidity rates associated to radical surgery or radiation (7). Specifically, AS is now a preferred option for many men with low-risk prostate cancer, gaining worldwide adoption due to robust data and is currently highlighted by many guidelines as the best treatment strategy for men with low risk (8, 9).

What constitutes the best approach to AS is an open question, as many protocols currently exists. However, to the patient selection questions, the field of urology sets the tone in low risk - PSA <10 ng/ml, WHO GG1 and a clinical stage T1c/T2a. There are several stricter protocols that have been developed and tested for AS. The Epstein criteria of ≤2 positive cores, <50% core involvement, and PSA density <0.15 ng/ml/cm^3^ carries 10 years rates of overall survival, cancer-specific survival, and metastasis-free survival of 94%, 99.9%, and 99.4%, respectively. Importantly, at 15 years, oncological outcomes such as metastasis-free survival and cancer specific survival change little (10).

In Canada, specifically Klotz and collaborators have reported on single-arm cohorts of low-risk patients (Gleason score ≤6 and serum PSA level ≤10 ng/mL) and favorable intermediate-risk patients (serum PSA ≤15 ng/mL or a Gleason score of 7 [3+4]). The investigators reported 10- and 15-year metastasis-free survival rates of 96% and 95% vs 91% and 82% for low vs. intermediate risk patients, respectively. Clearly the results are more consistent with low risk patients (11).

Nevertheless, a low-risk categorization does not equal to an indolent course. The presence of Gleason pattern 4 or 5 in patients diagnosed as low risk is about 30%, not insignificant (1). Importantly, as shown recently by Diamand et al., the upgrading phenomenon is persisting even as we trend into MR fusion biopsies. These researchers evaluated the accuracy in histologic grading of final histopathologic outcomes of radical prostatectomy specimens compared to systematic biopsies (SB), targeted biopsies (TB) and the combination of both (SB+TB). There was an upgrade by the final prostatectomy specimens of 43.1%, 39.5% and 23.9%, respectively (12).

Thus, the debate of AS is anything more intense and relevant than ever. As we described before, AS is particularly susceptible to perceptions and bias, life intricacies of both patients and their treating physicians (13). Compliance in AS demands a commitment, significant intellectualization, and conviction to ameliorate the anxiety motivated by thoughts, either own or from else, that the diagnosed cancer may mutate and go wild. In addition, the 2-year reclassification rates of patient in AS ranges from 49-64% (14). Thus, not uncommonly, many physicians have a low fuse for AS failure and recommend radical interventions. Not surprising, by 5 to 10 years after initiating AS more than 60% of patients change to radical management (15–19).

Perhaps the most critical caveat for AS and prostate cancer management in general is the black and white fixed view. We either treat the entire gland or we do not treat at all. This type of dilemma is pervasive to prostate cancer. In our urological field, we don't routinely dichotomize cancer management, perhaps the best example is bladder cancer management where 85% of patients are initially treated with a bladder preservation approach, as a diagnosis of high-risk transitional cell carcinoma does not equate to radical surgery. However, in prostate cancer the lack of color derives in our opinion from two consequential factors: 1 - How we make the diagnosis - *transrectal random* biopsy; and 2 - *Multifocal* biology of most prostate cancers. The former is by far where our patients and we could use some improvement (20). For starters, the random nature of the biopsy denies reliable precision. The transrectal approach is not applicable for optimal management.

The advance in prostate imaging has led to better recognition and imaging characterization of prostate cancer risk. MR fusion biopsies have emerged to deliver certainty on cancer location and extent. However, most of these techniques employ a transrectal technique, in our view a terrible flaw, and significant contributor to possible harm associated with prostate cancer diagnosis. However, there is an approach to sampling the prostate that is much safer, reliable and opens the door to partial prostate treatment: the transperineal approach. A few years ago, the concept of performing in office transperineal biopsies or treatments was considered non-sense or impossible. Currently, that is not the case; we have demonstrated this with over 1,015 of the procedures in the last 3 years performed using a local anesthesia block (21).

Thus, diagnosis prostate cancer with Transperineal MR Fusion provides for precise knowledge not just of location and extent, but to open the next frontier - the color box - for management: multifocal targeted partial gland ablation.

Some are calling “super-active surveillance” a partial gland ablation (22), we don't necessarily agree this might be the precise term. However, we do with the notion that alternatives are needed to fill the gap between the yin and yang - traditional AS vs. radical treatment. Today there are many energy options available to deliver partial cancer ablation, such as: cryoablation, high-intensity focused ultrasound (HIFU), radiofrequency ablation (RFA), laser ablation, irreversible electroporation (IRE), microwave ablation, photodynamic therapy and convective water vapor.

In our view, any attempt of intervention to the prostate must achieve the following characteristics: 1 - An extremely favorable adverse event profile, thus adverse events are rare, 2 - It must not burn any bridges, thus must not increase significantly the difficulty of radical surgery, shall the patient needed in the future and/or must increase adverse event profile of radiation, 3 - Convalescence from the procedure must be fast, carrying minimal interference with routine life activities, 4 - It should preserve prostate function, thus ejaculation shall be a measurable outcome as be erectile activity, and 5 - Urinary function shall exhibit improvement or no change, with no impact on urinary continence. When it comes to oncological outcomes, ablated cancer control must be demonstrated with tissue, we believe is imperative that patients receive a 1 year follow-up biopsy of ablated areas. Other oncological outcomes to consider include risk of conversion and freedom from androgen deprivation along with classic oncological measures as metastasis-fee survival, cancer specific and all cause survival. Currently, it's impossible to gauge all these outcomes and particularly difficult to compare ablation techniques, given the absence of high-quality and long follow-up studies in the literature (23–25). That is why we call this coming approach the next frontier.

Among the currently employed partial ablation techniques, cryotherapy and HIFU are the ones leading the race. Interestingly however, VTP is the only energy source studied in a multicenter, randomized, controlled phase III trial versus AS. VTP was superior in reducing presence of higher-grade cancer on biopsy (16% vs 41% Gleason 7), reducing chances of radical therapy (25).

Most ablation techniques focus in a zone, or area, some even consider the entire lobe. The argument against these approaches is raised by the multifocality of prostate cancer and what represents or conforms the index lesion. A notion that gas gained traction suggest that cancer related outcomes would derive from the index lesion and not by concomitant indolent tumors that may coexist at the time of diagnosis, as these are commonly low volume Gleason 3+3 (25, 26). Supporters of the index lesion highlight that these are the cancer lesions exhibiting highest Gleason scores and thus harbor incremental potential for local spread, recurrence, resistance and/or metastasis (27–30). However, certain ablation approaches such as Cryoablation and IRE portend advantages addressing multifocality making the theory of the index lesion irrelevant (20).

As mentioned before, the transperineal approach provides direct access to the prostate gland. No wonder the original radical prostatectomy began with a transperineal approach. However, the perineum is hindered by a perception of a need for general anesthesia. We would argue that the anesthesia need is dependent of the technique or energy to be used rather than by the anatomy. Since 2014, as we developed our technique to block the perineum we have performed 1,233 transperineal MR fusion prostate biopsies, and 578 transperineal MR fusion target prostate cancer cryoablations in the office setting under local anesthesia. Safety is the most significant advantage of this approach. We published some of our results and they show an adverse event profile under 3%, most low grade, such as urinary retention. Importantly, post biopsies or cryoablation admission are extremely rare (21).

Moreover, Bianco et al. recently provided with emerging data on MRI Fusion Target Cryoablation as a novel implementation of targeted partial gland ablation. The procedure was performed on 348 patients. Their median age was 71 and 218 had at least 1 year of follow-up. In this series, the median PSA decline was 30% and 164 patients had a follow up prostate fusion biopsy reportedly negative in 109 (66%). Of the positive biopsy patients, 38 were re-treated with 7 patients converted to surgery and 8 to radiation (31).

We have tested our transperineal MR Fusion approach with other energy devices such as needle RFA and circling RFA, while we have done so on limited numbers they seem to be well tolerated by patients and we envision a role in partial gland ablation. We have also tested IRE MR Fusion, however, it requires general anesthesia and that by it self put the energy device as does with HIFU at a different level in term of harms from general anesthesia.

We believe that what's being called “super-active surveillance” or our preference “precise multifocal partial gland ablation” is the next frontier for physicians and patients. The legacy of the randomized trials (AS vs. intervention) is our much profound understanding of the harms associated with intervention along with its limited benefits. Treating the cancer while preserving organ function if where the future is, AS is a good compromise given the impressions associated with traditional prostate cancer diagnosis, however, we must do better.

Technology advantages, along with critical thinking will serve as our fundamental tools to continue to advance in our quest to deliver better oncological and functional outcomes for our prostate cancer patients. Furthermore, the ablation immunology understanding is underway and preliminary evidence that supports prostate partial ablation vaccine potential by immune system boost has awarded 2018 American Urological Association best poster and warrants future investigations (32).

## References

[1] Garisto JD, Klotz L (2017). Active Surveillance for Prostate Cancer: How to Do It Right. Oncology (Williston Park).

[2] Loeb S, Bjurlin MA, Nicholson J, Tammela TL, Penson DF, Carter HB (2014). Overdiagnosis and overtreatment of prostate cancer. Eur Urol.

[3] Chou R, Croswell JM, Dana T, Bougatsos C, Blazina I, Fu R (2011). Screening for prostate cancer: a review of the evidence for the U.S. Preventive Services Task Force. Ann Intern Med.

[4] Wilt TJ, Jones KM, Barry MJ, Andriole GL, Culkin D, Wheeler T (2017). Follow-up of Prostatectomy versus Observation for Early Prostate Cancer. N Engl J Med.

[5] Bill-Axelson A, Holmberg L, Garmo H, Taari K, Busch C, Nordling S (2018). Radical Prostatectomy or Watchful Waiting in Prostate Cancer - 29-Year Follow-up. N Engl J Med.

[6] Donovan JL, Hamdy FC, Lane JA, Mason M, Metcalfe C, Walsh E (2016). Patient-Reported Outcomes after Monitoring, Surgery, or Radiotherapy for Prostate Cancer. N Engl J Med.

[7] Cerqueira MA de, Laranja WW, Sanches BC, Monti CR, Reis LO (2015). Burden of focal cryoablation versus brachytherapy versus active surveillance in the treatment of very low-risk prostate cancer: a preliminary head-to-head comprehensive assessment. Eur J Cancer Care (Engl).

[8] Sanda MG, Cadeddu JA, Kirkby E, Chen RC, Crispino T, Fontanarosa J (2018). Clinically Localized Prostate Cancer: AUA/ASTRO/SUO Guideline. Part I: Risk Stratification, Shared Decision Making, and Care Options. J Urol.

[9] Sanda MG, Cadeddu JA, Kirkby E, Chen RC, Crispino T, Fontanarosa J (2018). Clinically Localized Prostate Cancer: AUA/ASTRO/SUO Guideline. Part II: Recommended Approaches and Details of Specific Care Options. J Urol.

[10] Tosoian JJ, Mamawala M, Epstein JI, Landis P, Wolf S, Trock BJ (2015). Intermediate and Longer-Term Outcomes From a Prospective Active-Surveillance Program for Favorable-Risk Prostate Cancer. J Clin Oncol.

[11] Klotz L, Vesprini D, Sethukavalan P, Jethava V, Zhang L, Jain S (2015). Long-term follow-up of a large active surveillance cohort of patients with prostate cancer. J Clin Oncol.

[12] Diamand R, Oderda M, Al Hajj Obeid W, Albisinni S, Van Velthoven R, Fasolis G (2019). A multicentric study on accurate grading of prostate cancer with systematic and MRI/US fusion targeted biopsies: comparison with final histopathology after radical prostatectomy. World J Urol.

[13] Reis LO, Carter HB (2015). The Mind: Focal Cryotherapy in Low-Risk Prostate Cancer: Are We Treating the Cancer or the Mind?. Int Braz J Urol.

[14] Alam R, Carter HB, Landis P, Epstein JI, Mamawala M (2015). Conditional probability of reclassification in an active surveillance program for prostate cancer. J Urol.

[15] Musunuru HB, Yamamoto T, Klotz L, Ghanem G, Mamedov A, Sethukavalan P (2016). Active Surveillance for Intermediate Risk Prostate Cancer: Survival Outcomes in the Sunnybrook Experience. J Urol.

[16] Tosoian JJ, Mamawala M, Epstein JI, Landis P, Wolf S, Trock BJ (2015). Intermediate and Longer-Term Outcomes From a Prospective Active-Surveillance Program for Favorable-Risk Prostate Cancer. J Clin Oncol.

[17] Godtman RA, Holmberg E, Khatami A, Pihl CG, Stranne J, Hugosson J (2016). Long-term Results of Active Surveillance in the Göteborg Randomized, Population-based Prostate Cancer Screening Trial. Eur Urol.

[18] Klotz L, Zhang L, Lam A, Nam R, Mamedov A, Loblaw A (2010). Clinical results of long-term follow-up of a large, active surveillance cohort with localized prostate cancer. J Clin Oncol.

[19] Bul M, Zhu X, Valdagni R, Pickles T, Kakehi Y, Rannikko A (2013). Active surveillance for low-risk prostate cancer worldwide: the PRIAS study. Eur Urol.

[20] Reis LO, Billis A, Zequi SC, Tobias-Machado M, Viana P, Cerqueira M (2014). Supporting prostate cancer focal therapy: a multidisciplinary International Consensus of Experts (“ICE”). Aging Male.

[21] Bianco FJ, Grandez JA, Lozano S Transperineal procedures (biopsy and cryoablation) under local anesthesia: our experience after 1,015 procedures. The Journal of Urology.

[22] Bloom JB, Gold SA, Hale GR, Rayn KN, Sabarwal VK, Bakhutashvili I (2018). “Super-active surveillance”: MRI ultrasound fusion biopsy and ablation for less invasive management of prostate cancer. Gland Surg.

[23] Azzouzi AR, Vincendeau S, Barret E, Cicco A, Kleinclauss F, van der Poel HG (2017). Padeliporfin vascular-targeted photodynamic therapy versus active surveillance in men with low-risk prostate cancer (CLIN1001 PCM301): an open-label, phase 3, randomised controlled trial. Lancet Oncol.

[24] Rodriguez-Rivera JA, Rodriguez-Lay R, Zegarra-Montes L, Benzaghou F, Gaillac B, Azzouzi AR (2018). Expanding indication of padeliporfin (WST11) vascular-targeted photodynamic therapy: results of prostate cancer Latin-American multicenter study. Actas Urol Esp.

[25] Gill IS, Azzouzi AR, Emberton M (2018). Randomized Trial of Partial Gland Ablation with Vascular Targeted Phototherapy versus Active Surveillance for Low-risk Prostate Cancer: Extended Follow-up and Analyses of Effectiveness. J Urol.

[26] Villers A, McNeal JE, Freiha FS, Stamey TA (1992). Multiple cancers in the prostate. Morphologic features of clinically recognized versus incidental tumors. Cancer.

[27] Wise AM, Stamey TA, McNeal JE, Clayton JL (2002). Morphologic and clinical significance of multifocal prostate cancers in radical prostatectomy specimens. Urology.

[28] Sartor AO, Hricak H, Wheeler TM, Coleman J, Penson DF, Carroll PR (2008). Evaluating localized prostate cancer and identifying candidates for focal therapy. Urology.

[29] Bott SR, Ahmed HU, Hindley RG, Abdul-Rahman A, Freeman A, Emberton M (2010). The index lesion and focal therapy: an analysis of the pathological characteristics of prostate cancer. BJU Int.

[30] Liu W, Laitinen S, Khan S, Vihinen M, Kowalski J, Yu G (2009). Copy number analysis indicates monoclonal origin of lethal metastatic prostate cancer. Nat Med.

[31] Bianco FJ, Grandez JA, Lozano S (2018). Office-based MRI/US fusion target prostate cancer cryoablation under local anesthesia: 348 patients. J Urology.

[32] Cerqueira MA, Ferrari KL, Mattos AC de, Reis LO (2018). Local Immune Modulation By Decreasing Cd4+ / Cd8+ T Cells Ratio After Prostate Cancer Hemicryoablation. J Urol.

